# In the Caudal Pontine Reticular Nucleus, Kv1.1 Expression Is Soma Size Dependent and Invariant During Postnatal Development

**DOI:** 10.1002/cne.70119

**Published:** 2025-12-15

**Authors:** Justin Peterle, Kathrin Deborah Wicke, Christina Pätz‐Warncke, Felix Felmy

**Affiliations:** ^1^ Institute for Zoology University of Veterinary Medicine Hannover, Foundation Hannover Germany

**Keywords:** caudal pontine reticular nucleus, comparative anatomy, Kv1.1 channel subunit, medial nucleus of the trapezoid body, PnC giant neurons, postnatal development

## Abstract

The acoustic startle reaction is a rapid behavioral response to an unexpected auditory stimulus. In mammals, this reaction is based on an archaic reflex arch connecting auditory inputs via the sensor–motor interface in the caudal pontine reticular nucleus (PnC) of the reticular formation with motor output. The neuronal population in the PnC is heterogeneous, containing an interspersed set of giant neurons that represent the cellular substrate of the sensorimotor interface. To probe whether the heterogeneous cell population can be divided into distinct subpopulations based on somatic morphometry or potassium channel expression, we quantitatively analyzed immunofluorescence labeling in the PnC and compared this between three mammals. In gerbil, mouse, and Etruscan shrew, soma size and roundness showed a continuum over all analyzed cells. Somatic Kv1.1 labeling intensity continuously increased with increasing soma size. Overall, no subpopulations based on somatic morphometric parameters and Kv1.1 expression were observed, suggesting that the PnC is composed of neurons displaying a continuum of soma sizes. Moreover, in mice, neurons of the MNTB but not PnC showed a postnatal developmentally regulated Kv1.1 expression, contrasting this sensorimotor interface with the sensory nuclei of the auditory brainstem.

## Graphical abstract

The caudal pontine reticular nucleus (PnC) holds neurons of different functions and sizes. Our analysis shows that soma size and shape cannot distinguish between PnC subpopulations. Kv1.1 expression is scaled with the soma size. These findings are observed in three different mammals of different brain sizes, indicating a generalization. Moreover, the Kv1.1 scaling and expression are independent of postnatal development from P8 onward, distinguishing the PnC from auditory centers such as the medial nucleus of the trapezoid body.

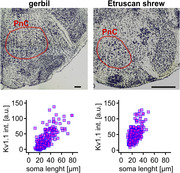

**Abbreviations**
ASR: acoustic startle reaction
Kv1.1: voltage‐gated potassium channel subunit 1.1
MAP2: microtubule‐associated protein 2
MNTB: medial nucleus of the trapezoid body
Mo5: trigeminal motor nucleus
NeuN: neuronal nuclear antigen
PBS: phosphate‐buffered saline
PFA: paraformaldehyde
PnC: caudal pontine reticular nucleus
VNLL: ventral nucleus of the lateral lemniscus

## 1 Introduction

The acoustic startle reaction (ASR) is an archaic reflex where an unexpected strong sound stimulation elicits a rapid muscular response (Koch and Schnitzler 1997). The ASR is found in all mammals and might be considered homologous in other vertebrate species (Zheng and Schmid 2023). The intensity and speed of the ASR increase with increasing loudness of the sound stimulus (Foss et al. 1989; Zheng et al. 2023). The neuronal circuit that mediates this behavioral response forms a short reflex arc, linking auditory nerve fibers and neurons in the cochlear nucleus to motor neurons via the caudal pontine reticular nucleus (PnC) of the reticular formation (Kandler and Herbert 1991; Lingenhohl and Friauf 1992, 1994; Lopez et al. 1999; Nodal and Lopez 2003). The sensorimotor interface is therefore established in the PnC.

The PnC consists of a population of neurons of varying sizes, including giant neurons. The range of soma sizes in the rat PnC is enormous, from small neuronal cell bodies (∼15 µm) to soma diameters in giant neurons of up to 100 µm (Andrezik and Beitz 1985; Lingenhohl and Friauf 1992; Newman 1985). In rats, a neuronal heterogeneous population with large somata between 35 and 80 µm is the cellular correlate of the sensory–motor interface of the ASR reflex (Kandler and Herbert 1991; Lingenhohl and Friauf 1992; Lopez et al. 1999; Nodal and Lopez 2003). It is unclear whether neurons with such somatic sizes are all and exclusively involved in the ASR or whether this functional group of neurons holds somatic distinctions from other PnC neurons. Moreover, the somatic morphology throughout the heterogeneous population of PnC neurons remains not fully quantified, and it is unclear to what extent the somatic morphology forms distinct subgroups or a continuum.

In rats, the neurons involved in ASR generate short latency excitatory postsynaptic potentials and rapid action potential responses to sound stimulations (Lingenhohl and Friauf 1992). The large size of neurons does not inherently support rapid signal output, because the time required to charge their membranes is longer compared to smaller neurons. To enable rapid output generation despite their large capacitance, the postsynaptic integration mechanisms might have to rely on voltage‐gated ion channels that speed up integrational properties of neurons.

Rapidly gating, low‐voltage‐activated potassium channels can mediate subthreshold coincidence detection by limiting the integration time of excitatory voltage responses (Mathews et al. 2010; Ovsepian et al. 2016; Scott et al. 2005). In the auditory systems, low‐voltage‐activated potassium channels have been shown to enhance timing and temporal precision of output generation (Dodson et al. 2002; Gittelman and Tempel 2006; Kopp‐Scheinpflug et al. 2003; Mathews et al. 2010; Rothman and Manis 2003; Scott et al. 2005; Svirskis et al. 2002) and are ideal candidates to set short integration time windows. In the auditory brainstem, low‐voltage‐activated potassium currents are often mediated by DTX‐sensitive Kv1 subunits (Cao and Oertel 2017; Dodson et al. 2002; Gittelman and Tempel 2006; Kladisios et al. 2023; Kopp‐Scheinpflug et al. 2003; Mathews et al. 2010; Scott et al. 2005; Svirskis et al. 2002). Since the startle reactions likely require coincidence integration of excitatory inputs in PnC neurons to generate reflex latencies within milliseconds (Ison et al. 1973), increased expression of the voltage‐gated potassium channel subunit 1.1 (Kv1.1) might compensate for the longer integration time caused by the large somatic capacitance in this sensory–motor interface.

In the PnC, at least giant neurons, or neurons larger than ∼35 µm, receive synaptic inputs originating from cochlear root neurons (Lingenhohl and Friauf 1992; Nodal and Lopez 2003; Simons‐Weidenmaier et al. 2006; Weber et al. 2002). Because these inputs also originate from the early central auditory system, and they also share the need for rapid synaptic transmission, PnC neurons might possibly receive comparable inputs to other rapidly integrating auditory brainstem nuclei. Auditory brainstem neurons undergo a large morphological and biophysical transition during postnatal development. Morphologically, changes in dendritic structure (Rautenberg et al. 2009; Rogowski and Feng 1981; Sanes et al. 1992), soma diameter (Franzen et al. 2015; Rogowski and Feng 1981), and axonal termination fields (Kim and Kandler 2003; Sturm et al. 2014; Werthat et al. 2008) are developmentally regulated. During the same period, the expression of voltage‐activated ion channels is altered, leading—for example, in the MSO—to an increase in DTX‐sensitive potassium currents (Scott et al. 2005). For sensory neurons of the auditory brainstem, these developmental refinements often occur before and after hearing onset and can be detected up to postnatal day (P) 27 (Ammer et al. 2012; Ford et al. 2009; Franzen et al. 2015; Rautenberg et al. 2009; Scott et al. 2005). Whether PnC neurons undergo similar postnatal developmental transitions in soma morphology or Kv1.1 expression as their auditory input structures is elusive.

The soma size of a neuron holds significant influence on its functional properties by affecting the membrane capacitance and often the input resistance, and its importance is highlighted by unexpected developmental alterations (Franzen et al. 2015). Moreover, the soma size of neurons usually scales with brain size (Beaulieu‐Laroche et al. 2021; Bekkers and Stevens 1990; Hardesty 1902). Since soma size in the PnC can be suggested to be functionally relevant, a comparative analysis of soma sizes could reveal the extent of scaling in this region and provide insights into its functional significance. Soma size in the rat PnC has been well established (Lingenhohl and Friauf 1992; Nodal and Lopez 2003). Therefore, to draw conclusions about PnC neuronal scaling, similar and differently sized mammals are required for comparison. Only slightly smaller mammals than rats are represented, for example, by gerbils. Since it is more reasonable to seek size differences by investigating smaller rather than larger mammals, the Etruscan shrew represents the ideal mammal. The Etruscan shrew is possibly the smallest terrestrial mammal, with a body size of 3–5 cm, a weight of 1.5–2.5 g, and a brain width that is about 0.4 times that of a gerbil.

To test whether, in general, sensory–motor neurons in the PnC can be segregated into distinct subpopulations based on somatic morphology or Kv1.1 expression, we carried out two different approaches. First, we comparatively analyzed the PnC somata in three different species and, second, throughout postnatal development. Moreover, as a sensory control nucleus, we applied the same analysis to the medial nucleus of the trapezoid body (MNTB). In none of the species (gerbil, mouse, Etruscan shrew) nor at any postnatal age could our analysis subdivide the large heterogeneity in the PnC into distinct subpopulations. However, the soma sizes appeared to scale with the brain and showed a developmental increase in the MNTB but hardly in the PnC. Kv1.1 expression levels scaled with soma size in the PnC and increased during postnatal development in the MNTB but not in the PnC of mice. The differences in developmental plasticity of the Kv1.1 expression between PnC and MNTB indicate that Kv1.1 may contribute to functions beyond the biophysical tuning of PnC neurons to auditory stimulation.

## 2 Materials and Methods

### 2.1 Animal Husbandry

Animals used in this study were housed and bred in the animal facility of the Institute of Zoology at the University for Veterinary Medicine, Hannover, or, in the case of mice, purchased at Charles River Laboratories. Gerbils of either sex between P43 and P50 (*N* = 6) and C57BL/6 mice of either sex aged P8 (*N* = 5), P10 (*N* = 4), P15 (*N* = 3), and P62–70 (*N* = 6) were used. Gerbils and mice were provided with food and water ad libitum. Etruscan shrews of either sex, in the age range between P138 and P223 (*N* = 7), were used and had access to water ad libitum. Etruscan shrews were fed with field crickets on a daily basis. Because of the small body size of Etruscan shrews, the number of sections obtained from one animal is low in juvenile animals. Therefore, older Etruscan shrews, in comparison to gerbils and mice, were used to obtain more sections from these animals and also to facilitate the preparation of the tissue. Since our own preliminary data show no age‐related hearing loss in Etruscan shrew, the data are considered comparable.

### 2.2 Animal and Tissue Preparation

Experiments were in agreement with local legislation of Lower Saxony and federal laws and approved by the local animal welfare officer with the number TiHo‐T‐2024‐3. Animals were euthanized with carbon dioxide and declared dead after a breathing arrest for more than 1 min. Animals were immediately transcardially perfused with heparinized phosphate‐buffered Ringer solution, and after blood drainage, perfusion was switched to 4% paraformaldehyde (PFA) for 15 min. Brains were removed and stored in 4% PFA overnight at 4°C. Brains were trimmed with a guillotine with an angle of 85° (Etruscan shrew) and 80° (gerbil, mouse). To prepare for cryosectioning, brains were incubated in 15% sucrose and thereafter in 30% sucrose, both at 4°C overnight. Sections with a thickness of 30 µm were intersected with the Mikrotom‐Kryostat HM 500 OM at an object and box temperature of −25°C and were stored in cryoprotective solution at 4°C (30% glycerol, 99% Sigma G9012‐1L; 30% ethylene glycol, 99% Roth 9516.5; 40% phosphate‐buffered saline [PBS]).

### 2.3 Immunofluorescence Labeling and Antibody Characterization

For standard Nissl staining, fixed sections containing the PnC region and the MNTB were mounted on glass slides, dried overnight, and thereafter stained with cresyl violet acetate (Roth) and sealed with DePex (Serva). For immunofluorescence labeling, sections were transferred into PBS and washed for 10 min with blocking solution (0.5% Triton, 1% bovine serum albumin, 0.1% saponin in PBS). Immunofluorescence labeling was performed by incubating sections in blocking solution containing the following primary antibodies (Kv1.1, rabbit, 1:2500, Alomone, Cat#APC‐009, RRID:AB_2040144; neuronal nuclear antigen (NeuN), mouse, 1:5000, Proteintech, Cat#66836‐1‐Ig, RRID:AB_2882179; microtubule‐associated protein 2 (MAP2), chicken, 1:3000, Neuromics, Cat#CH22103, RRID:AB_2314763) overnight at room temperature. Sections were washed three times for 10 min in blocking solution. Subsequently, sections were incubated in blocking solution containing the secondary antibodies (Cy3, anti‐rabbit, 1:800, Dianova, Cat#711‐165‐152, RRID:AB_2307443; Alexa488, anti‐mouse, 1:200/1:400, Dianova, Cat#715‐545‐150, RRID:AB_2340846; Alexa488, anti‐chicken, 1:300/1:800, Dianova, Cat#703‐546‐155, RRID:AB_2340376) at room temperature for 4 h. Sections were washed for 10 min in blocking solution and then four times for 10 min in PBS. Sections were mounted on gelatin‐covered slides with VECTASHIELD (Vector Laboratories) and sealed with nail polish. The samples were stored in the dark at 4°C.

Labeling of K_V_1.1 was performed with an antibody directed against the epitope with amino acid residues 416–495 of mouse K_V_1.1 and has been tested in mouse, rat, and human. It was tested and verified at a dilution of 1:200 in a knock‐out mouse (Zhou et al. 1998). Further, it was shown that K_V_1.1 labels SOC neurons of shrew and bat (Kladisios et al. 2023; Pätz et al. 2022; Zacher and Felmy 2024). Labeling of NeuN was performed with an antibody directed against the NeuN fusion protein Ag28016 (company description) of mouse. NeuN did not label Etruscan shrew neurons but successfully labeled somata in mice and gerbils (Keplinger et al. 2018; Pätz et al. 2018). Labeling of MAP2 was performed with an antibody directed against the recombinant human projection domain sequence, amino acids 377–1505 (company description). This antibody was tested in mouse, gerbil, human, and Etruscan Shrew (Couchman et al. 2010; Jacobs and Doering 2010; Lund et al. 2020; Zacher and Felmy 2024). Here, two combinations of antibodies were used: K_V_1.1 with NeuN and K_V_1.1 with MAP2.

### 2.4 Microscopy and Image Acquisition

Images were acquired with a confocal laser‐scanning microscope (Leica TCS SP5; Leica Microsystems GmbH, Wetzlar, Germany) with a 20x/0.7 NA objective, a sample speed of 400 Hz, and a 6x line average to acquire three consecutive images with a *z*‐step size of 0.17 µm, yielding a voxel size of 0.189 × 0.189 × 0.168 µm. The pixel number per line scan was set to 4096. Cy3 was excited at 561 nm and detected at wavelengths between 568 and 650 nm. Alexa488 fluorescence was excited at 488 nm and detected at wavelengths between 500 and 540 nm. The laser intensity and gain were kept constant for sections from a given animal to enable intensity comparison between cells. Between animals, only the gain of the photomultiplier tube was altered (10% of max) to detect Kv1.1 and NeuN equally well. To specify the presence of the PnC region in each section, the MNTB was used as the major landmark, together with the VII nerve. If the MNTB could not be captured together with the PnC region in one image, a second tile image was taken and combined post hoc.

### 2.5 Image Processing and Data Analysis

Color channels of K_V_1.1/NeuN and K_V_1.1/MAP2 immunofluorescence were combined in Fiji (Schindelin et al. 2012). For stitching tile images of the MNTB and PnC, a Fiji plugin (Preibisch et al. 2009) was used. Brightness and contrast were linearly adjusted to improve neuron detection. The settings were chosen equally for every section of a given animal to maintain comparability during data extraction. The extracted mean fluorescence value was independent of the brightness and contrast scaling and was based on the original microscope settings.

Individual neuronal somata were encircled with the freehand selection in Fiji (Schindelin et al. 2012) at a zoom of 150% for big (>30 µm soma length) and 200% for smaller somata (<30 µm soma length). All neuronal somata within a section were picked (mouse: P8 *n* = 218 from 23 sections, P10 *n* = 240 from 23 sections, P15 *n* = 220 from 13 sections; adult: *n* = 247 from 31 sections; shrew: *n* = 222 from 28 sections; gerbil: *n* = 226 from 19 sections). Somata were selected for encircling when (a) they showed no overlap with other neurons, (b) an apparent nucleus was visible due to the lack of Kv1.1 labeling, and (c) the soma labeling had sharp edges and was not blurred or merged with the background labeling. For mouse and gerbil, soma extraction was performed in the NeuN channel. Because the NeuN labeling for shrew did not work, the K_V_1.1 channel was used for the encircling of PnC and MNTB somata. Fluorescence mean intensity of somata was background‐corrected for every section. For background corrections, a circle with a diameter of 60 pixels was placed in an area with low fluorescence and no cell structure.

Somatic Feret length and angle, roundness, and soma width were analyzed. The Feret length and angle and soma area were extracted in Fiji. For the extracted Feret angle, the reference is the horizontal axis of the section. Soma roundness was calculated by dividing the measured Feret minimum by the Feret maximum length. The soma width was chosen as the major parameter for the cell size and was defined as the measured Feret maximum. Data were further analyzed in Microsoft Excel (Microsoft, Redmond, WA, USA) and Igor Pro 9 (Wavemetrics, Lake Oswego, OR, USA). Statistics were performed in R. The distribution of data was tested using the D'Agostino Normality test, testing for normality, skew, and kurtosis. To test for significant differences in pairwise comparisons of PnC and MNTB data, the Wilcoxon rank sum test was used, because all comparisons included at least one nonnormally distributed data point. Finally, for species and age comparisons, the Kruskal–Wallis rank sum test, together with the post hoc Wilcoxon rank sum test, was used to test for statistically significant differences.

## 3 Results

**FIGURE 1:**
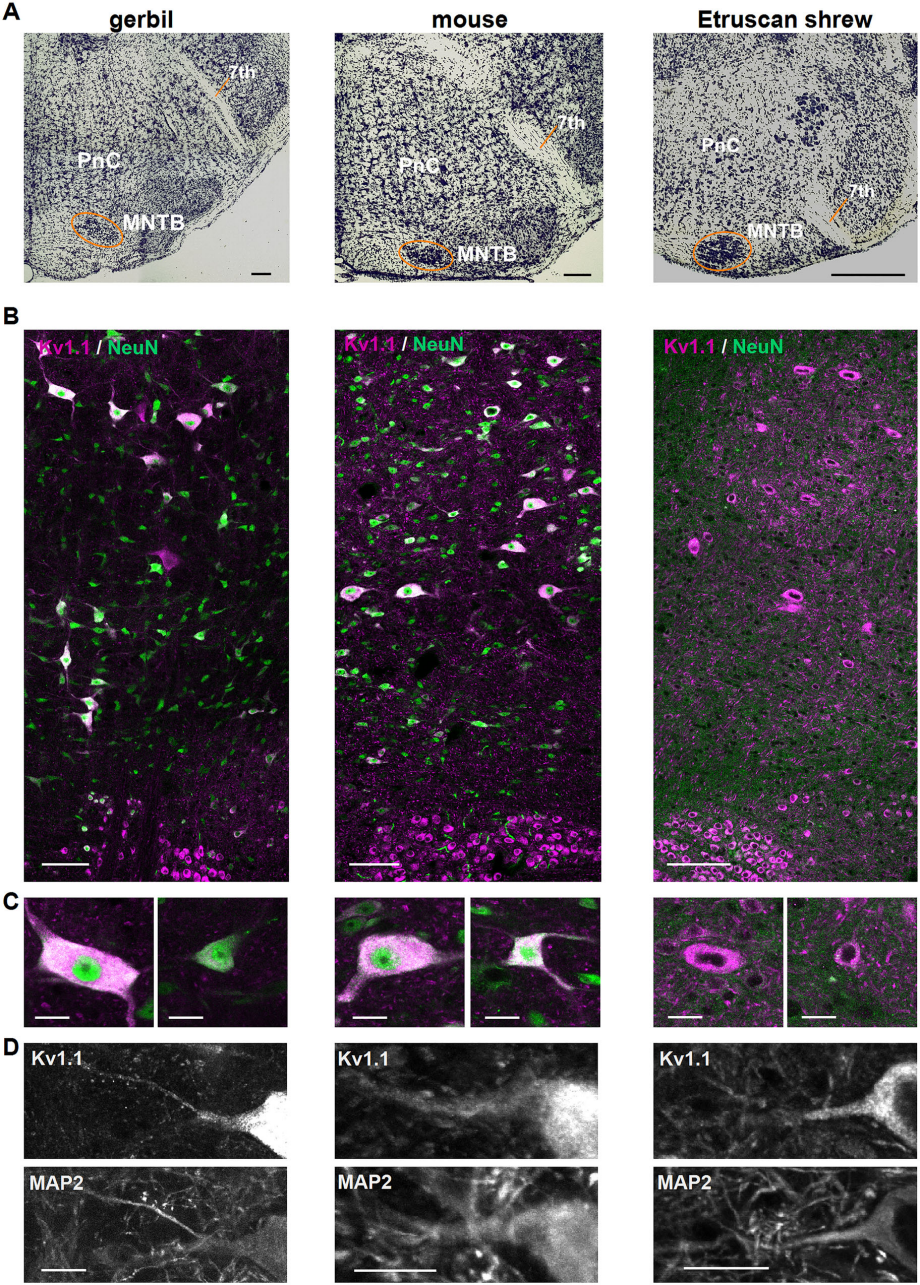
Kv1.1 expression indicates cellular heterogeneity in the PnC but not in the MNTB. (A) Nissl‐stained coronal overview sections containing the PnC region, MNTB, and seventh nerve in gerbil, mouse, and Etruscan shrew. Scale bars equal 200 µm. (B) Kv1.1 (magenta) and NeuN (green) labeling in the mature PnC and MNTB to detect neurons in gerbil, mouse, and Etruscan shrew. NeuN labeling failed to detect neurons in Etruscan shrew. Scale bars equal 100 µm. (C) Magnification of large and small Kv1.1‐labeled neurons in the PnC from gerbil, mouse, and Etruscan shrew. Same colors as in (A) and scale bars equal 20 µm. (D) Kv1.1 (top) and MAP2 (bottom) labeling of the soma and proximal dendrite, as well as small dendritic segments in mature PnC neurons in gerbil, mouse, and Etruscan shrew. Scale bar equals 20 µm.

In the three mammals—gerbil, mouse, and Etruscan shrew—the PnC appeared as a heterogeneous group of cells dorsal to the superior olivary complex, especially the MNTB (Figure 1). Besides the MNTB, the VII nerve or trigeminal motor nucleus (Mo5) was used as a second landmark, according to the literature (Allan Brain Atlas [https://atlas.brain‐map.org/atlas?atlas=1#atlas=1&plate=100960204&structure=313&x=5280&y=3745&zoom=‐4&resolution=16.75&z=5]; Radtke‐Schuller et al. 2016). The heterogeneous PnC cell population consisted of neurons with large and small somata (Figure 1). These neurons were successfully labeled for Kv1.1 (Figure 1B,C). In mature gerbils and mice, but not in Etruscan shrews, the cells expressing Kv1.1 could be co‐labeled with NeuN to prove their neuronal nature (Figure 1A,B) and additionally mark their somatic area. This co‐labeling suggests a size‐dependent Kv1.1 label intensity in the PnC (Figure 1B). The prominent somatic Kv1.1 labeling extends at least in the proximal dendrite of PnC neurons, as evident in the MAP2 co‐labeling (Figure 1D). From these labelings, it can also be derived that Kv1.1 is not present in the small dendritic segments and therefore most likely absent from distal processes.

Here, we investigate whether a distinct population based on somatic morphometry exists in the PnC. To do so, we extracted the cell diameter and area of each neuron soma irrespective of size from our immunofluorescently labeled sections. As a control population, the somatic parameters of MNTB neurons were collected. For mature gerbils and mice, the soma size estimate is based on NeuN fluorescence. Because NeuN labeling was unsuccessful in Etruscan shrews and NeuN fluorescence matches well in shape with the somatic Kv1.1 labeling in the other species, we used Kv1.1 labeling in Etruscan shews to extract the morphological parameters of the soma.

**FIGURE 2:**
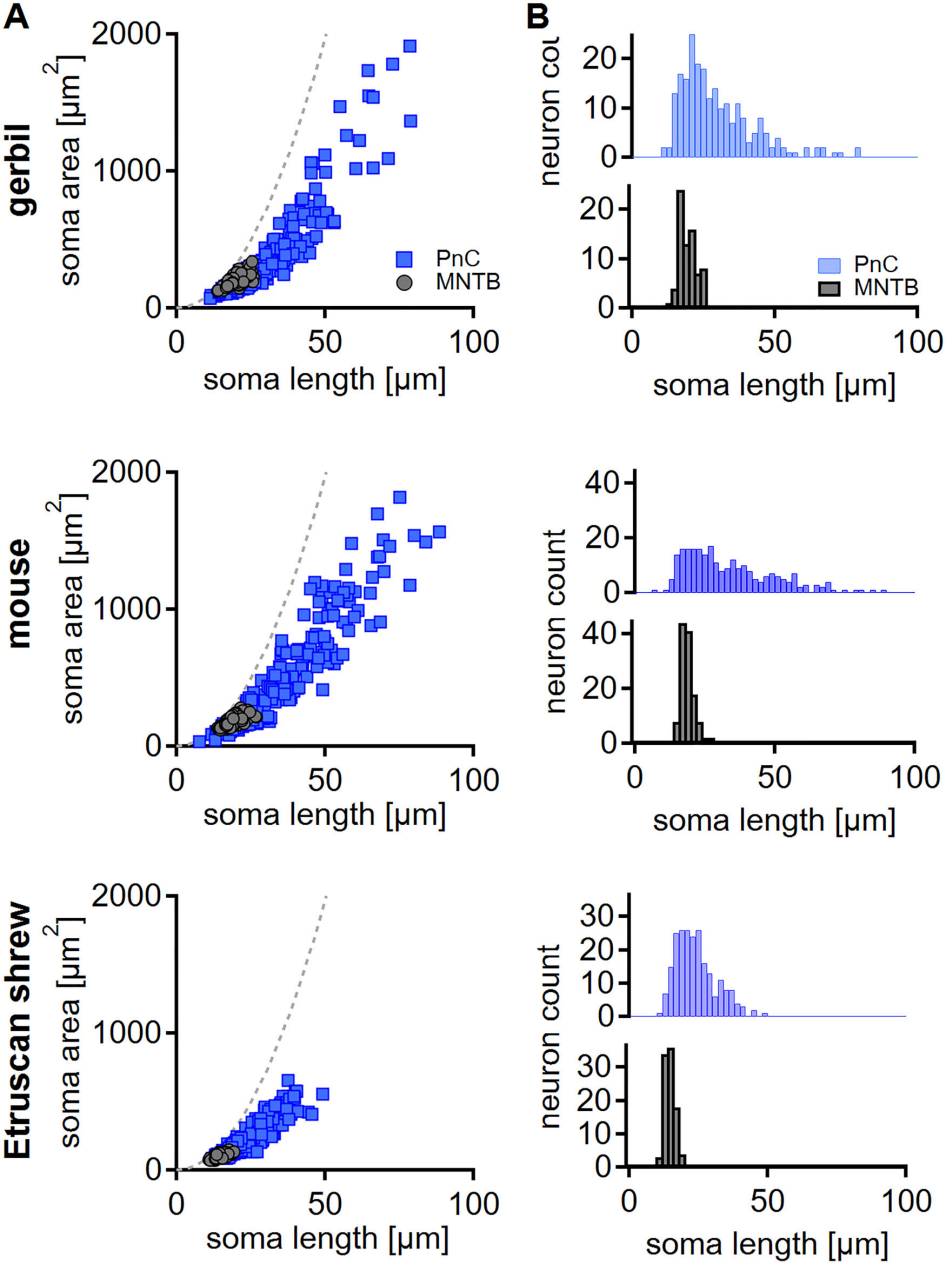
Distribution of soma sizes. (A) Quantification of maximal soma length and area from mature PnC (blue square) and MNTB (dark gray circle) neurons in gerbil, mouse, and Etruscan shrew (top to bottom). As a reference, the relation between the diameter and area of a circle is plotted as a dotted gray line. (B) Distribution of PnC (blue) and MNTB (black) soma length in the three given species indicates heterogeneous and homogeneous cell sizes, respectively.

The soma length correlated well with the soma area of PnC and MNTB neurons in all species tested (Figure 2A). With a larger soma length, however, the relative increase in area became smaller. The difference between length and area is predicted for cells that do not form perfect circles. Because soma length is commonly used as the describing parameter of PnC neurons in the existing literature (Andrezik and Beitz 1985; Lingenhohl and Friauf 1992; Newman 1985), it will be used as the relevant parameter in the following. In the PnC, the soma length is heterogeneous and shows a significant skew (gerbil: *χ* ^2^ = 56.5463, *p* = 5.26 × 10^−13^, skew = 6.6301, *p* = 3.35 × 10^−11^; mouse: *χ* ^2^ = 31.0154, *p* = 1.84 × 10^−7^, skew = 5.3982, *p* = 6.73 × 10^−8^; shrew: *χ* ^2^ = 21.0122, *p* = 2.74 × 10^−5^, skew = 4.4874, *p* = 7.21 × 10^−6^; D'Agostino Normality test; Figure 2B). The range of maximal soma length varies from 11.5 to 78.9 µm in gerbils, from 7.8 to 88.6 µm in mature mice, and from 11.9 to 49.3 µm in Etruscan shrews (Figure 2A–C). Statistically, Etruscan shrew PnC neurons are smaller compared to the other species (gerbil vs. shrew: *p* = 9.40 × 10^−6^; mouse vs. shrew: *p* = 2.10 × 10^−10^; Kruskal–Wallis test *p* = 1.34 × 10^−10^; post hoc Wilcoxon rank sum test). In comparison, the soma diameter of MNTB neurons showed no significant skew, or only a weak skew, and mostly followed a normal distribution (gerbil: *χ* ^2^ = 3.9934, *p* = 0.1358, skew = 1.4888, *p* = 0.1365; mouse: *χ* ^2^ = 17.6733, *p* = 0.0001453, skew = 3.4875, *p* = 0.0004875; shrew: *χ* ^2^ = 2.2617, *p* = 0.3228, skew = 1.479, *p* = 0.1391; D'Agostino Normality test; Figure 2B). In mature animals, the size difference between gerbil/Etruscan shrew and mouse/Etruscan shrew MNTB soma diameters was also significant (gerbil vs. shrew: *p* = 2.00 × 10^−16^; mouse vs. shrew: *p* = 2.00 × 10^−16^; Kruskal–Wallis test *p* = 2.20 × 10^−16^; post hoc Wilcoxon rank sum test) but not between gerbils and mice (gerbil vs. mouse: *p* = 0.12; Kruskal–Wallis test *p* = 2.20 × 10^−16^; post hoc Wilcoxon rank sum test). Taken together, the population of PnC neurons is heterogeneous in size, but a separation into distinct classes based on soma morphology is unfeasible, and soma size in small mammals partially scales with brain size.

**FIGURE 3:**
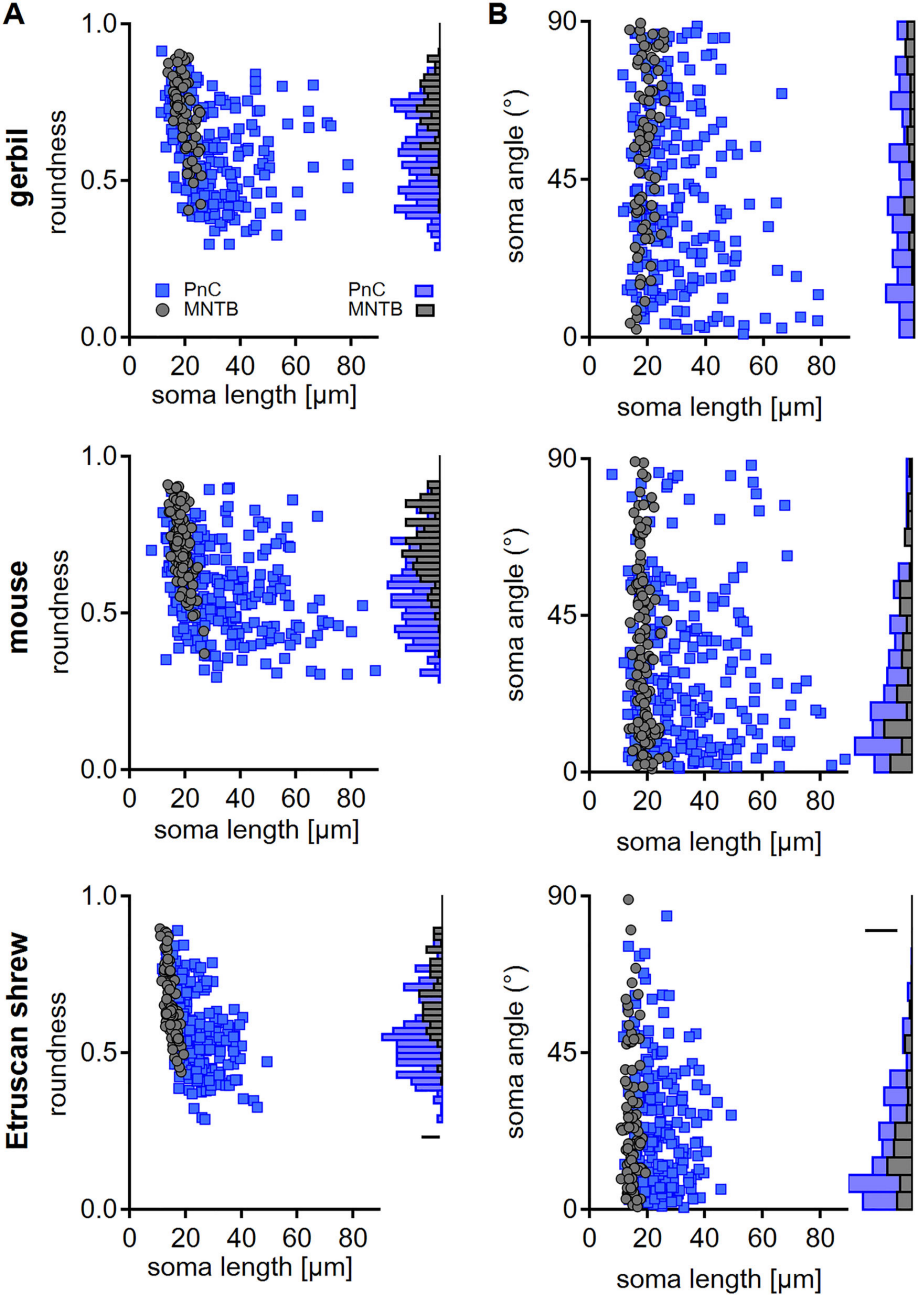
Heterogeneity of soma shape in the PnC forms a continuum. (A) Somata roundness for neurons of the mature PnC (blue square) and MNTB (dark gray circle) in gerbil, mouse, and Etruscan shrew (top to bottom) as a function of soma size. The right shows the roundness distribution of cells in the PnC (blue) and the MNTB (black). (B) Angle of the longest soma axis with respect to the horizontal axis (equals 0°) for neurons in the PnC (blue square) and MNTB (dark gray circle) as a function of soma size. The right shows the angle distribution of cells in the PnC (blue) and the MNTB (black).

To further seek the possibility of segregating neurons in the PnC into specific subclasses based on somatic morphology, we determined the roundness and angle of the neuronal somata. The roundness of PnC neurons indicated neurons from round to elongated morphology, while more round cells prevailed in the MTNB (Figure 3A). However, in mature animals, the roundness of the PnC neurons did not correlate with the size (gerbil: *p* = 0.0003178, *r* = −0.24; mouse: *p* = 6.66 × 10^−7^, *r* = −0.31; Etruscan shrew: *p* = 3.95 × 10^−12^, *r* = −0.44; Figure 3A). For MNTB neurons, the correlation between roundness and size was present in all species (gerbil: *p* = 2.36 × 10^−9^, *r* = −0.63; mouse: *p* = 2.06 × 10^−13^, *r* = −0.60; Etruscan shrew: *p* = 3.53 × 10^−16^, *r* = −0.72; Figure 3A,C,E). Despite these missing or present correlations, the distributions of roundness did not suggest specific subclasses of neurons in the PnC or MNTB. Overall, in all species, PnC neurons showed a larger heterogeneity in their shape, with more elongated cells compared to MNTB somata.

The orientation of the somata determined by the Feret angle between the soma's longest axis and the horizontal axis showed no correlation with the soma size (Figure 3B) in any species in the mature PnC (gerbil: *p* = 0.856, *r* = −0.01; mouse: *p* = 0.257, *r* = 0.07; Etruscan shrew: *p* = 0.148, *r* = 0.10) or MNTB (gerbil: *p* = 0.593, *r* = 0.06; mouse: *p* = 0.4134, *r* = 0.07; Etruscan shrew: *p* = 0.7798, *r* = −0.03). However, there appear to be species‐specific differences in the distributions of angles in these nuclei. In gerbils, orientations appeared with no preference, while in mice and in Etruscan shrew, the distributions showed a significant skew to horizontally orientated cells (gerbil: skew = 0.8078, *p* = 2.20 × 10^−16^; mouse: skew = 5.3495, *p* = 5.61 × 10^−7^; shrew: skew = 5.3316, *p* = 2.16 × 10^−7^; D'Agostino Normality test; Figure 3B). In the mature MNTB, the orientation showed no differences between gerbils and mice, and no specific orientation was detectable, while in Etruscan shrews, a more mediolateral orientation was found (Figure 3B). Taken together, somatic morphometry does not allow for a clear segregation of specific neuronal cell types within the PnC, because the somata in this region form a continuum of shapes and sizes. Furthermore, the orientation of neuronal somata within a given brain area appears to be species‐dependent.

**FIGURE 4:**
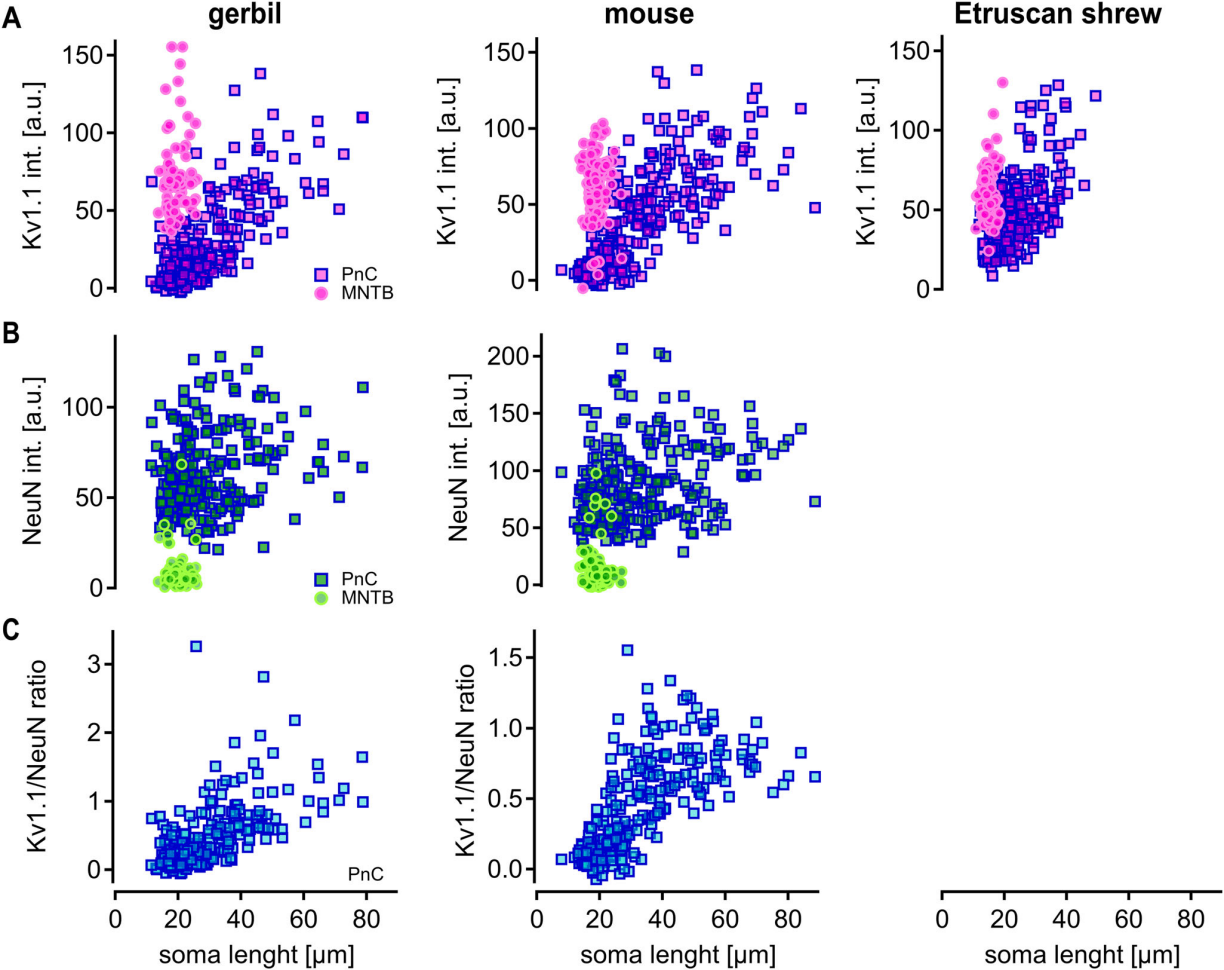
Kv1.1 expression increases with soma length in PnC neurons. (A) Somatic Kv1.1 labeling intensity (magenta) as a function of soma length for mature PnC (square) and MNTB (circle) neurons in gerbil, mouse, and Etruscan shrew. (B) Somatic NeuN labeling intensity (green) as a function of soma length for PnC (square) and MNTB (circle) neurons. (C) Ratio of Kv1.1 and NeuN labeling intensity for PnC neurons. Note that due to the lack of NeuN‐specific staining, no NeuN or ratio data can be presented for Etruscan shrew.

Next, we quantified the mean fluorescence of the Kv1.1‐labeled soma with respect to soma size in the PnC and MNTB. Kv1.1‐mediated currents can limit integration times (Mathews et al. 2010; Scott et al. 2005), thereby compensating for the effects of large soma sizes, which suggests that Kv1.1‐mediated currents may be particularly critical in large neurons of the PnC. In mature animals, Kv1.1 intensity was low in small neurons and higher in larger neurons of the PnC. Background‐corrected Kv1.1 labeling intensity correlated in all tested species with the soma size of PnC neurons (gerbil: *p* = 2.20 × 10^−16^, *r* = 0.74; mouse: *p* = 2.20 × 10^−16^, *r* = 0.75; Etruscan shrew: *p* = 2.20 × 10^−16^, *r* = 0.56) but not MNTB neurons (gerbil: *p* = 0.5662, *r* = 0.07; mouse: *p* = 0.414, *r* = 0.07; Etruscan shrew: *p* = 0.02335, *r* = 0.23; Figure 4A). Only in large PnC neurons did the intensity of Kv1.1 labeling match that in MNTB somata. However, no clear subpopulation of specifically Kv1.1‐labeled neurons emerged. To ascertain that in PnC neurons the increase in mean Kv1.1 fluorescence intensity reflects an increase in expression level and not only soma size, we also analyzed the mean fluorescence intensity of the structural marker NeuN. Since the structural markers’ mean intensity should not be correlated with the size of the soma, it serves as an internal control. Indeed, no correlation between NeuN label ing and soma length was observed in gerbils or mice (Figure 4A,B) for neither PnC neurons (gerbil: *p* = 0.0002838, *r* = 0.24; mouse: *p* = 1.32 × 10^−5^, *r* = 0.27) nor MNTB neurons (gerbil: *p* = 0.9429, *r* = −0.01; mouse: *p* = 0.7337, *r* = 0.03). To corroborate the size‐dependent changes in Kv1.1 expression in PnC neurons, we normalized Kv1.1 by the NeuN fluorescence. This analysis shows that the Kv1.1/NeuN ratio increases with increasing soma length (Figure 4C) in gerbils (*p* = 2.20 × 10^−16^, *r* = 0.60) and mice (*p* = 2.20 × 10^−16^, *r* = 0.68). Thus, larger PnC neurons contain more Kv1.1 channels not only because of their size but also due to relatively higher expression levels. However, this size‐dependent increase in expression did not allow for specifying a distinct subpopulation of PnC neurons. The size‐dependent Kv1.1 expression was found in all investigated mammalian species with up to 94 MYA evolutionary segregation time (http://www.timetree.org/).

**FIGURE 5:**
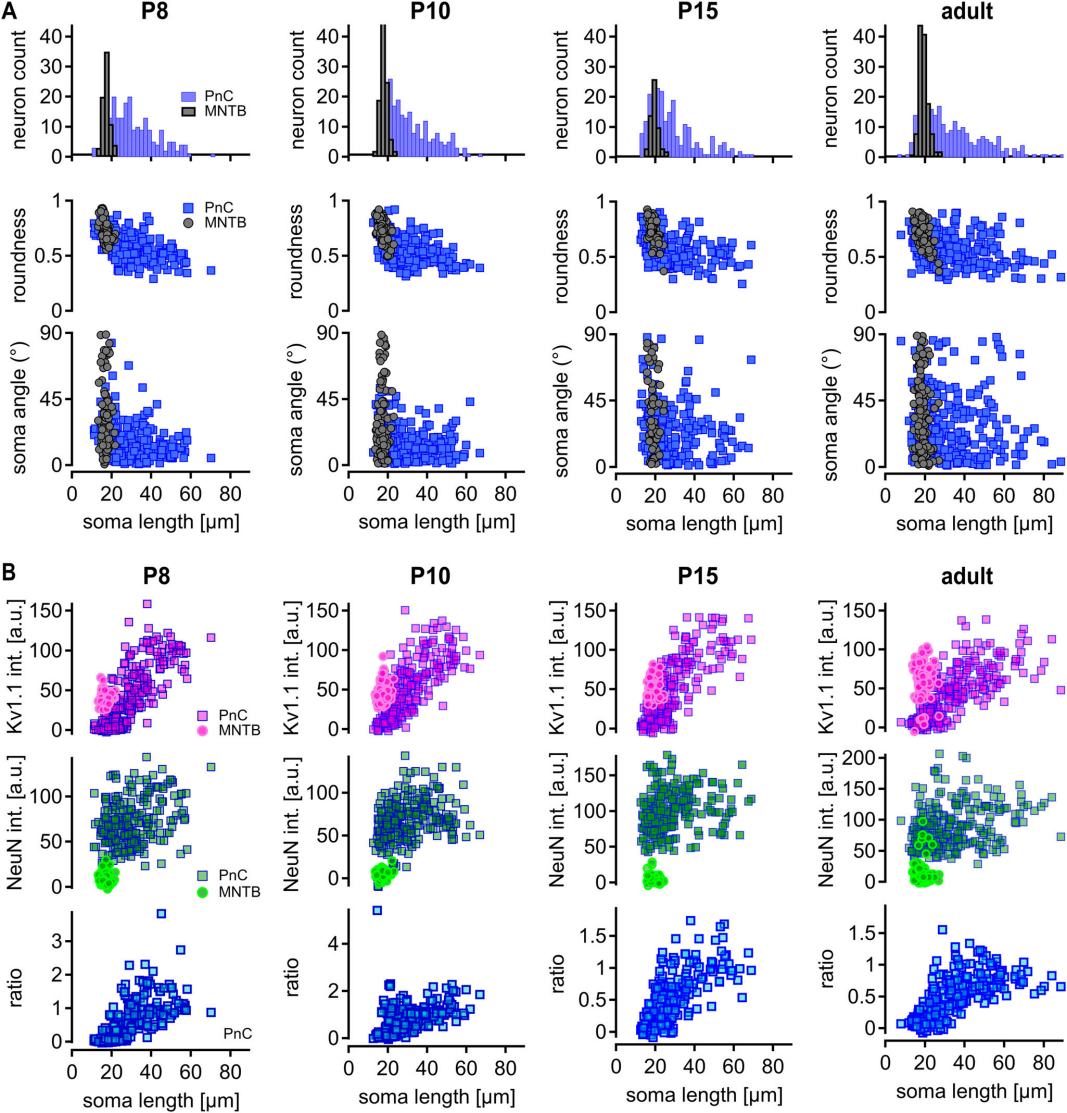
Developmental profile of soma length and Kv1.1 expression in mouse PnC and MNTB. (A) Somatic morphology of PnC (blue) and MNTB (black) neurons in developing mice. From left to right in P8, P10, P15, and adult mice. Top: soma size distribution; middle: soma roundness; and bottom: soma angle. (B) Somatic Kv1.1 expression of PnC (square) and MNTB (circle) neurons in developing mice. From left to right in P8, P10, P15, and adult mice. Top: Kv1.1 intensity (magenta) for PnC (square) and MNTB (circle) neurons; middle: NeuN intensity (green) for PnC (square) and MNTB (circle) neurons; and bottom: ratio between Kv1.1 and NeuN fluorescence for PnC neurons.

In addition to the analysis of different mammals, the developmental profile of PnC soma morphology and Kv1.1 expression was analyzed in mice between P8 and the mature form. The distribution of soma sizes in the PnC showed in all age groups a skew to larger values (P8: skew = 4.0924, *p* = 0.0002265; P10: skew = 4.5278, *p* = 2.77 × 10^−5^; P15: skew = 5.9242, *p* = 3.93 × 10^−9^; mature: skew = 5.3982, *p* = 1.84 × 10^−7^; D'Agostino Normality test; Figure 5A). This skew becomes slightly larger in adult animals by extending to neurons with diameters between 60 and 80 µm (Figure 5A), while the distribution itself did not change significantly during postnatal development (P8 vs. adult: *p* = 0.209; P10 vs. adult: *p* = 0.0131; P15 vs. adult: 0.012; P8 vs. P10: *p* = 0.608; P8 vs. P15: *p* = 0.117; P10 vs. P15: *p* = 0.192; Kruskal–Wallis test *p* = 0.01291; post hoc Wilcoxon rank sum test; Figure 5A). The lack of change in size distribution in the PnC differs from the MNTB where cells tend to increase in size over postnatal development (P8 vs. adult: *p* = 9.40 × 10^−9^; P10 vs. adult: *p* = 2.40 × 10^−6^; P15 vs. adult: *p* = 0.491; P8 vs. P10: *p* = 0.072; P8 vs. P15: *p* = 9.40 × 10^−9^; P10 vs. P15: *p* = 2.60 × 10^−6^; Kruskal–Wallis test *p* = 6.02 × 10^−13^; post hoc Wilcoxon rank sum test; Figure 5A) without introducing a specific skew (P8: skew = 1.7203, *p* = 0.1069; P10: skew = 1.8975, *p* = 0.104; P15: skew = 2.1197, *p* = 0.08337; mature: skew = 3.4875, *p* = 0.0001453; D'Agostino Normality test; Figure 5A) or a change in roundness (*p* = 0.7573; Kruskal–Wallis test; Figure 5A).

The background‐corrected Kv1.1 intensity did not show a substantial increase during the different age groups in mice PnC neurons (*p* = 0.08967; Kruskal–Wallis test) and stretched over a large range of values in a size‐dependent manner (Figure 5B). However, in the MNTB, the Kv1.1 intensity showed a smaller variance compared to the PnC and increased from P8 to adult animals (*p* = 2.20 × 10^−16^, Kruskal–Wallis test; P8 vs. adult: *p* = 4.70 × 10^−16^; P10 vs. adult: *p* = 2.20 × 10^−13^; P15 vs. adult: *p* = 0.0082; Figure 5B). To corroborate the specificity of the Kv1.1 intensity, NeuN values were again extracted. In PnC and MNTB, no change in NeuN fluorescence intensity was observed during development (Figure 5B). For PnC neurons, we calculated again the Kv1.1/NeuN intensity ratio and found that no substantial changes occurred during development and that the soma size‐dependent fluorescence increase was present from P8 onward (P8: *p* = 2.20 × 10^−16^, *r* = 0.63; P10: *p* = 6.16 × 10^−15^, *r* = 0.48; P15: *p* = 2.20 × 10^−16^, *r* = 0.71; Figure 5B). Together, these findings indicate that the Kv1.1 function is profound and has a purpose beyond age‐related biophysical tuning toward sound stimulations.

## 4 Discussion

The large heterogeneity of the somatic morphologies of PnC neurons is common to all three mammalian species tested in this study, where soma sizes span over a large range (10–80 µm) with different shapes and orientations. Based on somatic morphometric features, no subdivision of the neuronal populations can be proposed in the PnC. Thus, PnC neurons form a large heterogeneous continuum of somatic morphologies. During postnatal development in mouse, an apparent increase in soma size for large neurons is suggested. Most somata in the PnC area express the Kv1.1 subunit, with the expression strength depending on soma size. This latter finding shows no developmental modulation from P8 onward in mice. Overall, the data of the PnC contrast the somatic morphometry and Kv1.1 expression in MNTB neurons, where a rather homogeneous group of neurons with small‐sized, roundish somata increases the expression of Kv1.1 channels and soma size during development in mice.

In mammals, neuronal soma size scales with brain size (Beaulieu‐Laroche et al. 2021; Bekkers and Stevens 1990; Hardesty 1902). In the cortex, for example, not only the length of the apical dendrite but also the soma size increases (Beaulieu‐Laroche et al. 2021). This increase in soma size is also observed in the PnC neuronal population when comparing the distributions between Etruscan shrew, the smallest terrestrial mammal, mouse, and gerbil. Similarly, the MNTB neurons are the smallest in Etruscan shrew. Yet, the brain size‐dependent scaling in the PnC and MNTB is not strict, because mice and gerbils appear to have similarly sized neurons despite slight differences in brain size. Whether this similarity is a possible exception, or a rodent or functional adaptation, remains unclear. With respect to the comparative approach, we note that Kv1.1 expression appears highly conserved. The size‐dependent level of Kv1.1 was found in mammals that segregate over 94 MYA and therefore likely reflects the general pattern in the PnC.

Within the auditory brainstem, neuronal soma size can be developmentally regulated (Franzen et al. 2015; Rogowski and Feng 1981), as in the ventral nucleus of the lateral lemniscus (VNLL), a reduction in diameter occurs in the late postnatal period. Such somatic changes can influence the input–output function of neurons and are therefore physiologically relevant (Bekkers and Stevens 1990; Franzen et al. 2015). In the PnC, the size distribution was largely unchanged, but a small fraction of the largest neurons appeared to emerge during development. Also, in the MNTB, the soma size increased during development in mice. Thus, soma size changes during development might be rather ubiquitous and can occur in different directions. The soma size increase in PnC and MNTB indicates an increase in membrane capacitance and likely in membrane time constant, thereby suggesting a slowed integration of synaptic inputs. It is especially interesting to note the developmental somatic size increase in the MNTB because in the VNLL—another auditory nucleus innervated with a large somatic excitatory terminal—the somatic size becomes reduced during development (Franzen et al. 2015). Thus, it can be speculated that MNTB and VNLL neurons might generate their temporally precise output based on different cellular parameters and support the idea that, in general, the soma size development is crucial for the appropriate biophysical tuning of neurons.

We observed a difference in somatic angle with respect to the horizontal axis in the different species examined. In mice and Etruscan shrews, especially larger somata appeared more elongated and oriented toward the horizontal axis, while gerbil somata were rounder, independent of their size. Since we took care to sample from the same region with respect to the superior olivary complex, the VII nerve, and Mo5, we consider the region homologous between the investigated species. However, a clear limitation of this approach is that the PnC has not been mapped in detail in each species. Therefore, differences in the functional area or subnucleus cannot be fully ruled out. This limitation is especially unfortunate, as in rats, slightly more elongated neurons have been described in regions of the reticular formation that are adjacent to the PnC (Andrezik and Beitz 1985; Newman 1985). Assuming the correct location, our data show that the somatic morphology based on size, shape, and orientation angle does not allow for segregating PnC neurons into distinct subclasses. Therefore, PnC giant neurons cannot be morphologically classified per se, and no specific cutoff to smaller‐sized PnC neurons can be applied. The lack of morphological segregation does not exclude that functional subgroups are present in this brainstem area. Especially, as the PnC has been implied in other functions beyond the startle reaction, among such are sleep and eye movements (Cohen and Komatsuzaki 1972; Homma et al. 2002; Peter et al. 2008; Schmid et al. 2003; Shook et al. 1990; Yeomans et al. 2002). However, these functional subgroups might be defined by specific protein expression patterns or synaptic connectivity and could still have specific soma sizes.

As shown here, PnC neurons are heterogeneous regarding their soma size and shape. The Kv1.1 subunit expression level appears to match the soma size heterogeneity. Small somata are, on average, nearly devoid of Kv1.1, while large somata have high levels inferred from strong fluorescence labeling. This difference in labeling intensity could not be used to segregate specific PnC cell populations, because the continuum of intensities was present, as for the soma size. Thus, other ion channels or cell markers might be relevant for segregating specific PnC neuronal subpopulations and possibly illustrate their size dependency, in case subpopulations in the PnC exist.

In large PnC neurons, the labeling intensity exceeded that from MNTB neurons, a type of neuron known for its substantial Kv1.1 low‐voltage‐activated potassium current (Dodson et al. 2002). This suggests that large PnC neurons generate a strong low‐voltage‐activated potassium current. Moreover, our analysis suggests that an increase in low‐voltage‐activated potassium currents with increasing soma size might compensate for the capacitance‐dependent increase in membrane time constant. Assuming that in PnC neurons the Kv1.1‐mediated current also activates close to resting levels, as in other brainstem neurons (Mathews et al. 2010; Myoga et al. 2014; Scott et al. 2005), these channels would decrease the membrane input resistance, leading, on one hand, to an increase in rheobase current thresholds. On the other hand, the larger capacitance values would compensate for the lower input resistances, leading to a soma‐size‐independent membrane time constant, thus equalizing subthreshold integration times across soma sizes. Biophysically, this would mean that independent of soma size, PnC neurons would integrate slow inputs over a similar time course due to the normalization of the membrane time constant. Thus, the voltage response to slower synaptic or modulatory inputs might be very similar between differently sized PnC neurons. Contrary, very fast inputs of below 5% of the membrane time constant that are integrated largely by the capacitive element, corresponding to the soma size (Franzen et al. 2015), will therefore be integrated in a different manner between PnC neurons. Thus, for rapid synaptic excitation, differences in soma size will lead to different current thresholds. Taken together, this would generate a soma‐size‐dependent filter bank for rapidly evoked action potentials while equalizing the effect of slower modulatory inputs.

In mice, Kv1.1 subunits were expressed in the same soma size dependency over the postnatal developmental period investigated. Since hearing onset in mice is around P11, the expression level of Kv1.1 subunits is not affected by the opening of the ear canal and hence sound experience. This developmental independence is in contrast to the expression level of Kv1.1 channels in the primary auditory brainstem pathways, where an upregulation occurs at least in the MSO (Scott et al. 2005) and is likely relevant for other auditory brainstem nuclei that rely on low‐voltage‐activated potassium currents (Cao and Oertel 2017; Dodson et al. 2002; Rothman and Manis 2003). This developmental increase in Kv1.1 is also apparent in our labeling in the MNTB. Thus, the brainstem neurons tuned especially for sound processing are developmentally regulated differently from the sensory–motor interface of the ASR. Furthermore, the lack of developmentally regulated Kv1.1 in the PnC might suggest that this subunit plays a basic and fundamental role throughout life. This might include voltage signaling or other functional aspects. Alternatively, it might indicate that either there are no substantial developmental changes after P8, the Kv1.1 subunit is not an appropriate indicator for developmental regulation in PnC neurons, or the upregulation might happen earlier than P8, as the integration of the tactile modality might precede sound‐evoked activity.

## Author Contributions

**Justin Peterle**: data acquisition, data analysis, manuscript editing. **Kathrin Deborah Wicke**: data analysis, manuscript editing. **Christina Pätz‐Warncke**: data acquisition, manuscript editing. **Felix Felmy**: study design and concept, data acquisition and analysis, writing.

## Funding

This work was supported by the University of Veterinary Medicine Hannover, Foundation.

## Conflicts of Interest

The authors declare no conflicts of interest.

## Data Availability

The data that support the findings of this study are available from the corresponding author upon reasonable request.
